# A SENP7-SIRT1-IL-10 Axis Driven by DeSUMOylation Promotes Breg Differentiation and Immune Evasion in Colorectal Cancer

**DOI:** 10.7150/ijbs.118896

**Published:** 2026-01-01

**Authors:** Yuhan Liao, Xinghua Zhuo, Yuan Huang, Huimeng Xu, Zhe Hao, Lanhui Huang, Haoxuan Zheng, Jun Zhou

**Affiliations:** 1 Department of Pathology, Nanfang Hospital, School of Basic Medical Sciences, Southern Medical University, 1838 Guangzhou North Road, Guangzhou, Guangdong, 510515, China.; 2 Guangdong Province Key Laboratory of Molecular Tumor Pathology, Guangzhou, Guangdong, 510515, China.; 3 Guangdong Provincial Key Laboratory of Gastroenterology, Department of Gastroenterology, Nanfang Hospital, Southern Medical University, Guangzhou, 510515, China.; 4 Department of Pathology, The Tenth Affiliated Hospital (Dongguan People's Hospital), Southern Medical University, 78 Wandao Road, Dongguan, 523039, China.; 5 Dongguan Clinical Pathology Diagnosis Center, 14 Yuhua Road, Dongguan, 523001, China.; 6 Dongguan Key Laboratory of Clinical Pathology, 14 Yuhua Road, Dongguan, 523001, China; 7 Department of Urology, Nanfang Hospital, Southern Medical University, Guangzhou, 510515, China.

**Keywords:** sentrin-specific protease 7 (SENP7), regulatory B cells (Bregs), NAD-dependent protein deacetylase sirtuin-1 (SIRT1), DeSUMOylation, senescence, tumour microenvironment (TME)

## Abstract

Colorectal cancer (CRC) poses a significant global health challenge, yet immune checkpoint blockade (ICB) therapy benefits only a small subset of patients with mismatch repair-deficient (dMMR) or microsatellite instability-high (MSI-H) tumours. Through analyses of public single-cell and spatial transcriptomic datasets, primary mouse cell sorting and adoptive transfer experiments, flow cytometry, multiplex immunofluorescence, immunohistochemistry, and coimmunoprecipitation, we revealed that sentrin-specific protease 7 (SENP7) promotes regulatory B-cell (Breg) differentiation and inhibits senescence by activating the expression of the NAD-dependent protein deacetylase sirtuin-1 (SIRT1) via deSUMOylation, thereby enhancing the expression of genes such as interleukin-10 (IL-10). Notably, targeting SENP7 in B cells improved the antitumour efficacy of anti-PD-1 therapy. These findings suggest that inhibiting SENP7 may offer a promising strategy to sensitize immunologically “cold” tumours to immune checkpoint blockade.

## Introduction

Colorectal cancer (CRC) is a major global health threat and ranks as the third most commonly diagnosed cancer worldwide^1^. In recent years, immune checkpoint blockade (ICB) therapy has markedly improved treatment outcomes in multiple cancer types^2^-^7^. Despite its clinical benefits, ICB therapy is effective mainly in patients with metastatic colorectal cancer (mCRC) with deficient DNA mismatch repair (dMMR) or high microsatellite instability (MSI-H), representing only approximately 15% of all CRC cases^8^. One major challenge in ongoing research is broadening the effectiveness of immunotherapy to benefit a relatively large proportion of patients with CRC.

Abnormal immune activity in the tumour microenvironment (TME) is frequently linked to the presence of exhausted T cells (Tex), which, although still alive, show impaired effector capabilities^9^. Exhausted T cells are characterized by elevated levels of various coinhibitory receptors, with PD-1 being the most notable, along with molecules such as LAG3, TIM3, and TIGIT.

Regulatory B cells (Bregs) represent a distinct B lymphocyte subset that secretes multiple anti-inflammatory cytokines, including interleukin-10 (IL-10), IL-35, and transforming growth factor-β (TGF-β). Breg-derived IL-10 acts as a key immunosuppressive mediator that inhibits CD8⁺ T-cell activity in the TME, thereby promoting immune escape^10^.

Sentrin-specific protease 7 (SENP7), a SUMO-specific protease, cleaves SUMO modifications from target proteins, thereby influencing their stability and functional activity in tumour cells^11^. For example, in prostate cancer, degradation of the deSUMOylase SENP7 promotes SUMOylation of HP1α, thereby inducing cellular senescence and exerting tumour-suppressive effects^12^. NAD-dependent protein deacetylase sirtuin-1 (SIRT1) is an NAD⁺-dependent deacetylase whose enzymatic activity can be augmented by SUMOylation, and it plays dual roles in tumour suppression and oncogenesis^13^. Moreover, SIRT1 regulates IL-10 production at the transcriptional level^14^,^15^, and the loss or inactivation of SIRT1 accelerates senescence^16^-^18^. SUMO-specific proteases, including SENP1, directly remove SUMO modifications from SIRT1, thereby regulating its substrate deacetylation capacity, such as that of p53, under stress conditions^19^. By analogy, SENP7 may similarly target SIRT1 and thereby influence cancer progression and therapy.

This study revealed that targeting SENP7 in B cells by disrupting its regulatory influence on the SIRT1/IL-10 axis attenuated Breg-mediated immunosuppression and restored CD8⁺ T-cell functionality. Specifically, this work centers on the SENP7-SIRT1-IL-10 axis as the conceptual core, elucidating how deSUMOylation-driven modulation of B cell senescence and IL-10 production governs immune suppression and responsiveness to immunotherapy in colorectal cancer. When combined with anti-PD-1 therapy, SENP7 inhibition synergistically enhances antitumour immune responses. These findings provide a rationale for B-cell-targeted modulation of SENP7 as a novel combinatorial strategy to increase the clinical benefit of immunotherapy in CRC.

## Materials and Methods

### Data source and preprocessing

Single-cell RNA sequencing (scRNA-seq) data were obtained from the GSE178341, GSE161277, GSE205506, and GSE236696 datasets (GEO database). Spatial transcriptomic sequencing data were sourced from the GSM7089856 dataset (GEO database). The RNA-seq and clinical data were accessed via the TCGA database. The pancancer proteomic profiles were downloaded from the National Cancer Institute's Clinical Proteomic Tumour Analysis Consortium (CPTAC) Pan-Cancer Proteome release (https://pdc.cancer.gov/pdc/cptac-pancancer). The proteomic profiles (n=1,119), which included 11 tumour types—BRCA, ccRCC, COAD, GBM, HGSC, HNSCC, LSCC, LUAD, PDAC, UCEC and MB—were harmonized and uniformly processed by the Broad Institute with Spectrum Mill (PMID: 37582358). Genes associated with cellular senescence were obtained from the CellAge database (The Database of Cell Senescence Genes)^20^, as detailed in Supplementary Table 3. For the scRNA-seq data, batch effects were removed using the Harmony R package. Raw scRNA-seq data were filtered to remove low-quality cells (<200 genes, >10% mitochondrial content) and normalized using Seurat's LogNormalize method. Spatial transcriptomic data were preprocessed using Seurat's standard pipeline, including normalization and scaling.

### Single-cell analysis

Dimensionality reduction and clustering were carried out using the R package Seurat. Batch effects were removed with the Harmony algorithm, using patient ID as the batch variable. Uniform manifold approximation and projection (UMAP) was applied to identify cell clusters. Cell types were annotated using the R package SingleR. Senescence inhibition and induction scores were computed for each B cell using gene signatures from the CellAge database with Seurat's AddModuleScore function, and scores were projected onto UMAP plots with density-based visualization. Spatial transcriptomic data were analysed in Seurat to assess the colocalization of SENP7, IL10, and CD20.

### Pathway enrichment analysis

For KEGG pathway enrichment, differentially expressed genes (DEGs) between Bregs and other B cells were identified using Seurat's FindMarkers function (Wilcoxon rank-sum test, adjusted *p* < 0.05, |log2FC| > 0.25). DEGs were subjected to KEGG pathway analysis using the R package clusterProfiler. For hallmark-based GSEA, SENP7 expression data from the TCGA-CRC cohort were analysed using the R package fgsea. Genes were ranked by their correlation with SENP7 expression (Pearson correlation), and enrichment in hallmark gene sets from MSigDB. The enriched pathways are listed in Supplementary Table 4.

### Pan-cancer proteomic analysis

Correlation analysis was performed on CPTAC proteomic data to identify proteins positively correlated with SENP7 (correlation coefficient >0.2 and *p* <0.001) using the cor.test function in R. A total of 317 proteins positively correlated with SENP7 were identified, as detailed in Supplementary Table 5.

### Molecular docking analysis

Molecular docking was performed to predictt protein‒protein interactions between SENP7 and candidate proteins (POLR2A, CDKN2AIP, SIRT1, GABPB1, and TBX21) using AlphaFold3 (https://alphafoldserver.com). For every pair, a top-ranked complex was generated and evaluated with two AlphaFold confidence metrics: the predicted TM-score (pTM), which reflects the accuracy of the complex's overall fold (0-1, higher = better), and the interface-predicted TM-score (ipTM), which gauges the precision of the binding interface itself (0-1, higher = better). In this study, the combined value ip^TM^ + p^TM^ was used as an integrated indicator of interaction likelihood.

### Patient samples

This study was approved by the Institutional Review Board of The Tenth Affiliated Hospital of Southern Medical University (Dongguan People's Hospital). Colorectal cancer (CRC) patient samples were obtained from individuals who received immune checkpoint inhibitor (ICI) therapy prior to surgical resection at The Tenth Affiliated Hospital of Southern Medical University (Dongguan People's Hospital) between September 2019 and September 2023 (n = 45). Patients were categorized into two groups based on postoperative tumour regression grade (TRG): pathological complete response (pCR, n = 20) and non-pCR (n = 25). The efficacy of ICI treatment for CRC was evaluated using the College of American Pathologists Tumor Regression Grading (CAP-TRG) system. This grading system defines TRG0 as complete tumour regression with no viable cancer cells; TRG1 as near-complete response with only scattered single cells or small clusters remaining; TRG2 as partial response where residual tumour exceeds fibrosis; and TRG3 as poor or no response with extensive viable tumour. For analytical purposes, TRG0 was classified as pCR, whereas TRG1-3 were grouped as non-pCR.

### Isolation of splenic and tumour-derived cells

To generate single-cell suspensions, mouse spleens and tumour tissues were collected in DMEM supplemented with 10% foetal bovine serum (FBS). The tumours were finely chopped and enzymatically digested in DMEM containing 0.5 mg/mL collagenase IV and 0.1 mg/mL DNase I (Sigma‒Aldrich) at 37 °C for 1 hour with continuous agitation. This was followed by mechanical dissociation using frosted glass slides. The cell mixtures were then passed through a 70 μm filter (Solarbio Life Science) to remove debris. This protocol supports downstream applications such as cytokine profiling and exhaustion marker assessment of CD8⁺ T cells, depending on the experimental objectives.

Spleens were processed similarly by mechanical dissociation, but without enzymatic treatment. The resulting suspensions were filtered through 70 μm strainers. Red blood cells were removed by incubating spleen suspensions with 2 mL RBC lysis buffer (Leagene) for 2 minutes at room temperature, followed by neutralization with DMEM. The isolated splenocytes were subsequently used for sorting CD8⁺ T cells.

### Flow cytometry and cell sorting

Immune cells were resuspended in staining buffer and pretreated with Fc receptor blocking reagent (BioLegend) for 10 minutes. Surface markers were subsequently stained using fluorophore-conjugated antibodies for 30 minutes at 4 °C in the dark. For intracellular staining, the cells were fixed and permeabilized with Fixation/Permeabilization Concentrate and Diluent (eBioscience, mixed at a 1:3 ratio) for 30 minutes, followed by two washes with 1× Permeabilization Buffer. Intracellular antibodies were then added, and the samples were incubated under the same conditions. After staining, the cells were analysed using a BD flow cytometer. To detect intracellular cytokines, cells were stimulated with a cell activation cocktail containing brefeldin A (BioLegend) for 4-6 hours prior to staining.

CD8⁺ T cells and B cells were isolated by magnetic-activated cell sorting (MACS) using mouse-specific CD8⁺ T-cell and B-cell isolation kits (STEMCELL Technologies), respectively.

### Coculture of CD8⁺ T cells with Bgc cells

Mouse CD8⁺ T cells were purified via MACS and maintained in RPMI-1640 medium supplemented with 10% foetal bovine serum (FBS). T-cell activation was induced using anti-CD3 (2 μg/mL) and anti-CD28 (5 μg/mL) antibodies for 72 hours. Separately, B cells were also isolated by MACS and then cocultured with preactivated CD8⁺ T cells at a 1:2 ratio for 48 hours. Following coculture, cytokine secretion and exhaustion marker expression in CD8⁺ T cells were evaluated by flow cytometric analysis.

### *In vivo* B-cell adoptive transfer experiment

On Day 0, MC38 cells (5 × 10⁵) were subcutaneously injected into C57BL/6 or muMt^-/-^ mice. The next day, spleen-derived B cells (5 × 10⁵ per mouse) were intravenously injected into the tumour-bearing mice. A second B-cell transfer was performed on Day 7 following tumour implantation using the same administration route.

### Mice

MuMt^-/-^ (C57BL/6J) and wild-type (WT) mice were obtained from Zhaoqing Huaxia Kaiqi Biotechnology. In this mouse model, exon 2 was targeted for deletion using the gRNA sequences GTGTTCGTCCCACCACGGGA and CAGCCAGTCGATTTCAGAGA, resulting in a 197 bp deletion fragment. The PCR primers used for Ighm-KO genotyping in muMt^-/-^ mice are listed in Supplementary Table S1.

All animal experiments in this study were approved by the animal ethics committee of the Tenth Affiliated Hospital of Southern Medical University (Dongguan People's Hospital) (approval number: IACUC-AWEC-202506018) and conducted under SPF conditions in accordance with approved protocols. The tumour size was measured using a Vernier calliper twice per week, and the tumour volume (mm^3^) was calculated by the following equation: tumour volume = length × (width)^2^/2.

### Tumour growth and treatment

For the subcutaneous xenograft model, MC38 colorectal cancer cells (1 × 10⁶ per mouse) were injected subcutaneously to establish solid tumours. After 19 days, the mice were euthanized via carbon dioxide (CO₂) asphyxiation, and the tumours were excised for histological analysis.

In specific experiments, CD8⁺ T cells were depleted using anti-mouse CD8 antibodies (200 μg per mouse, administered twice per week), and PD-1 signalling was inhibited using anti-mouse PD-1 antibodies at the same dosage and frequency. Appropriate isotype control antibodies were used in parallel for both the CD8 and PD-1 intervention groups.

### Multiplex immunofluorescence staining

FFPE tissue sections (4 μm) were deparaffinized, rehydrated, and subjected to antigen retrieval in citrate buffer (pH 6.0) using a pressure cooker. After blocking with 3% BSA for 30 min at room temperature, primary and HRP-conjugated secondary antibodies were sequentially applied, followed by TSA fluorophore development (PerkinElmer). Antibody stripping between cycles was performed using a microwave in citrate buffer. The nuclei were counterstained with DAPI, and the slides were mounted with antifade medium.

Cells were fixed in 4% paraformaldehyde for 15 min, permeabilized with 0.2% Triton X-100 for 10 min, and blocked with 5% BSA for 1 h. Primary antibodies were incubated overnight at 4 °C, followed by incubation with fluorophore-conjugated secondary antibodies for 1 h at room temperature in the dark. After DAPI staining, the samples were mounted with antifade reagent.

Fluorescence imaging was performed using a Leica TCS SP8 confocal microscope or a Pannoramic MIDI II slide scanner (3DHISTECH) for whole-slide acquisition.

## Results

### Characterization of Bregs in CRC using single-cell RNA sequencing

**Figure 1:**
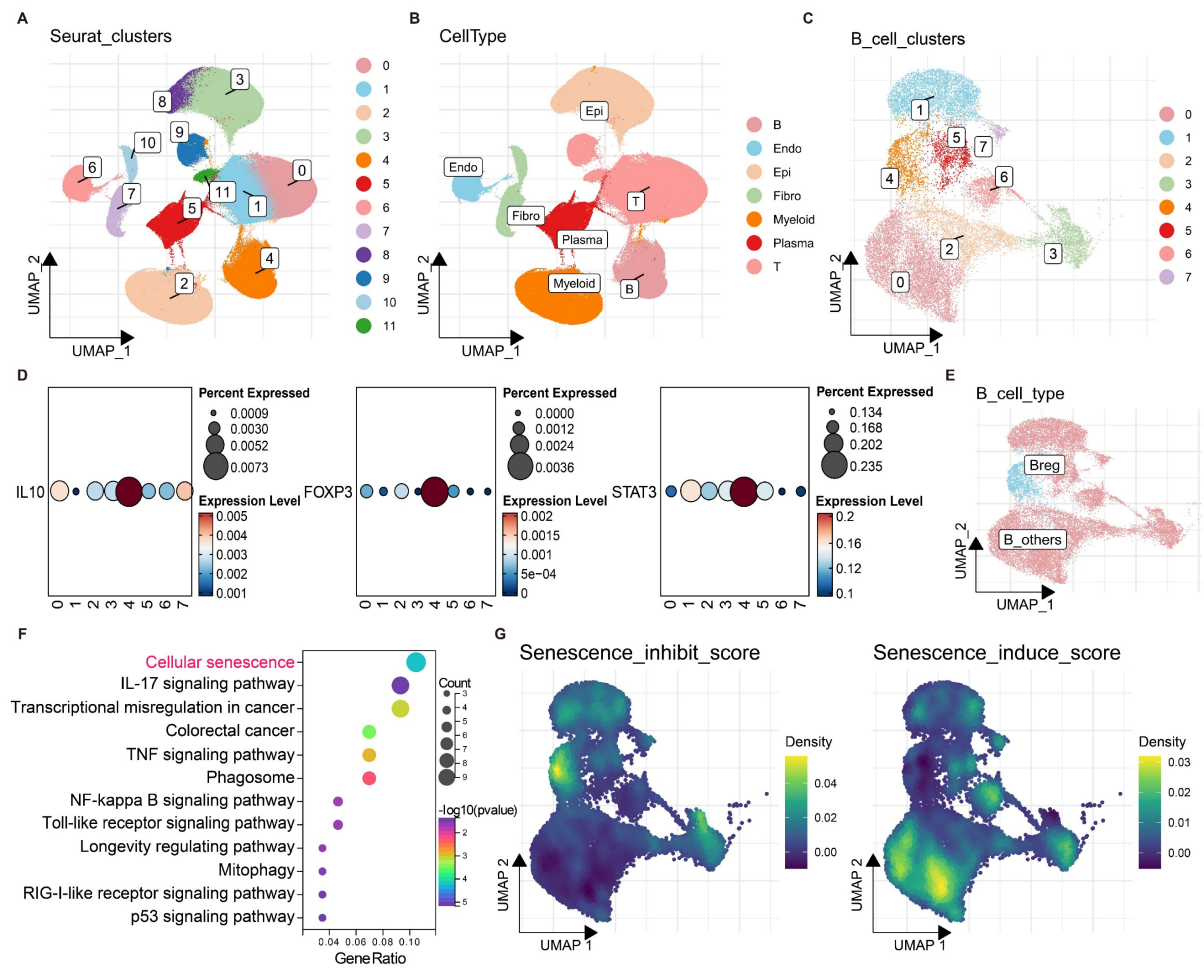
** Characterization of B regulatory cells (Bregs) in colorectal cancer (CRC) using single-cell RNA sequencing. (A)** UMAP plot of CRC single-cell data after batch effect removal, coloured by Seurat clusters (0-11). **(B)** UMAP visualization of annotated cell types, including epithelial (Epi), immune (T, B), myeloid (Myeloid), and stromal (Fibro, Endo) cells. **(C)** UMAP plot of B cells clustered into eight distinct groups (0-7).** (D)** Expression levels of Breg marker genes (IL10, FOXP3, and STAT3) across B-cell clusters, with percentages expressed indicated by dot size and expression level by colour intensity (0-0.235). **(E)** UMAP plot distinguishing Bregs (cluster 4) from other B cells (B_others).** (F)** KEGG pathway enrichment analysis of genes highly expressed in Bregs, showing significant enrichment in cellular senescence and IL-17 signalling pathways.** (G)** UMAP plots of B cells displaying senescence inhibition scores (left) and senescence induction scores (right), with density represented by a colour gradient (0-0.5). Compared with other B cells, Bregs exhibit the highest senescence inhibition scores and the lowest senescence induction scores.

To identify B regulatory cells (Bregs) in colorectal cancer (CRC), batch effects were first removed from 4 CRC single-cell datasets (Fig. S1A, B), followed by dimensionality reduction and clustering (Fig. 1A). A single-cell atlas of CRC was constructed, in which epithelial cells (Epi), immune cells (T, B), myeloid cells (Myeloid), and stromal cells (Fibro, Endo) were identified (Fig. 1B). B cells were subsequently extracted for redimensionality reduction and clustering, which revealed 8 distinct B-cell clusters (0-7) (Fig. 1C).

The expression levels of Breg markers, including IL10, FOXP3, and STAT3, were found highly expressed in the B-cell cluster 4 (Fig. 1D). In addition, the Breg signatures reported by Zhou *et al.* (2024)^21^ also exhibited a distinct enrichment in the B-cell cluster 4 (Fig. S1C, D). Therefore, B-cell cluster 4 was identified as the subpopulation with the most prominent Breg characteristics and was thus designated Bregs; the remaining B cells were classified as B_others (Fig. 1E).

Notably, genes highly expressed in Bregs were significantly enriched in KEGG pathways related to cellular senescence and IL-17 signalling, suggesting a potential role for cellular senescence in Breg differentiation (Fig. 1F). Furthermore, senescence inhibition and induction scores were calculated for each B cell and projected onto the B-cell atlas. Compared with other B cells, Bregs exhibited the highest senescence inhibition scores and the lowest senescence induction scores (Fig. 1G). These findings strongly suggest that the suppression of cellular senescence may promote Breg differentiation.

### Role of SENP7 in Breg differentiation and cellular senescence in CRC

**Figure 2:**
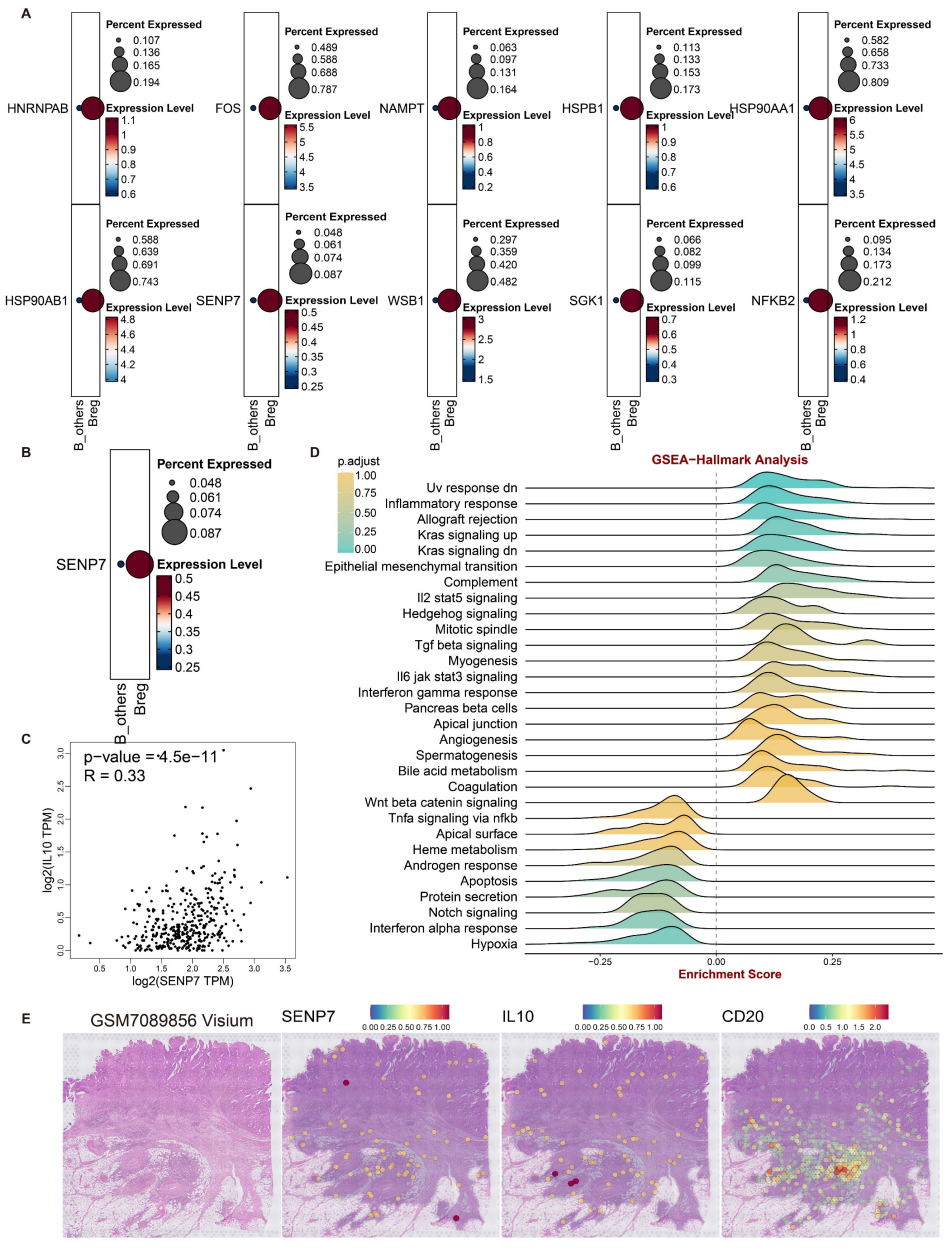
** Role of SENP7 in Breg differentiation and cellular senescence in colorectal cancer (CRC). (A)** Dot plots showing the percent expression and expression levels of ten senescence-related genes upregulated in Breg cells compared with other B cells, namely, HNRNPAB, FOS, NAMPT, HSPB1, HSP90AA1, HSP90AB1, SENP7, WSBI, SGK1, and NFKB2. **(B)** Kaplan-Meier survival curves for the TCGA-CRC cohort stratified by SENP7 expression (high vs. low), with log-rank P = 3.5e-02. **(C)** Scatter plot of SENP7 TPM (transcripts per million) versus IL10 TPM (transcripts per million), showing a significant correlation (R = 0.33, P = 4.5e-11). **(D)** GSEA of SENP7 expression. **(E)** Visualization of the spatial transcriptomic dataset GSM7089856 from CRC tissue reveals the colocalization of SENP7, IL10, and CD20, with expression levels represented by colour intensities ranging from 0-2.0.

To investigate how inhibition of cellular senescence induces Breg differentiation, the senescence-suppressing genes were projected onto B cells. The results revealed that 10 senescence-suppressing genes were specifically upregulated in Bregs (Fig. 2A). Kaplan‒Meier (KM) survival analysis of the TCGA-CRC cohort revealed that only sentrin-specific protease 7 (SENP7) was significantly associated with poor prognosis and positively correlated with interleukin-10 (IL-10) expression in patients with CRC (Fig. 2B, C). These findings suggested that SENP7 likely promoted Breg differentiation by suppressing B-cell senescence and upregulating IL10 expression. GSEA enrichment analysis of SENP7 indicated its potential involvement in activating the TGFβ signalling pathway (Fig. 2D), further strengthening the link between SENP7 and IL10.

Additionally, analyses of 3 CRC spatial transcriptomics datasets revealed a high degree of spatial colocalization among SENP7, IL-10, and CD20 (Fig. 2E & Fig. S2A-R), suggesting a potential spatial association between SENP7-expressing B cells and IL-10 production within the tumor microenvironment. However, we acknowledge that spatial proximity does not necessarily indicate direct molecular interaction or paracrine crosstalk, and further functional validation is required to confirm this relationship. These findings strongly supported the hypothesis that SENP7 might induce Breg differentiation by promoting IL10 secretion.

### SENP7 promotes Breg differentiation and inhibits Breg cell senescence

**Figure 3:**
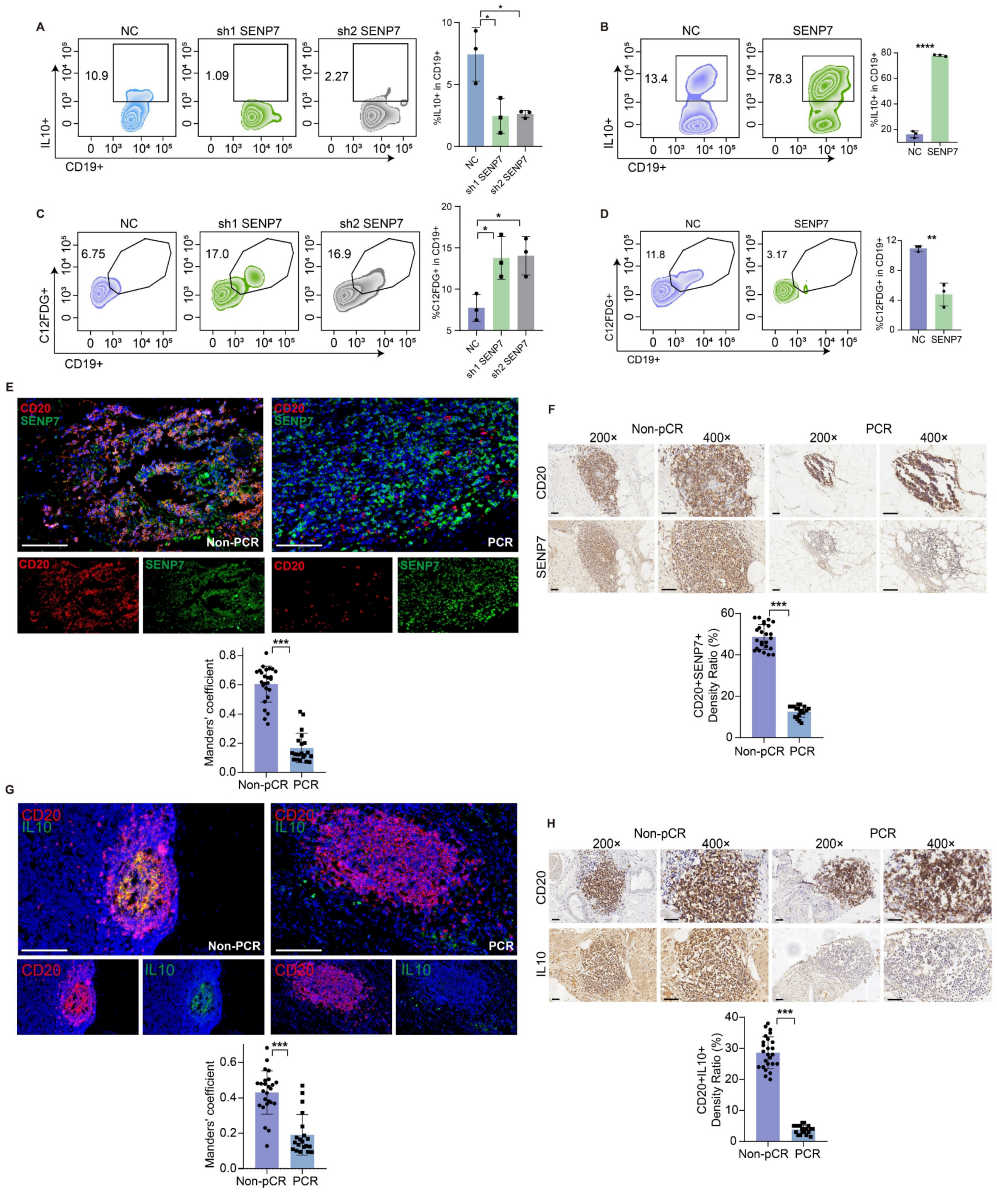
** SENP7 promotes Breg differentiation and inhibits Breg cell senescence. (A)** Representative flow cytometry plots and quantification of IL-10⁺-expressing primary mouse B cells following SENP7 knockdown compared with those following control treatment. **(B)** Representative flow cytometry plots and quantification of IL-10⁺-overexpressing primary mouse B cells following SENP7 overexpression compared with control cells. **(C)** Representative flow cytometry plots and quantification of C₁₂FDG⁺ primary mouse B cells following SENP7 knockdown compared with those following control treatment. **(D)** Representative flow cytometry plots and quantification of C₁₂FDG⁺ primary mouse B cells following SENP7 overexpression compared with those following control treatment. **(E)** Representative multiplex immunofluorescence staining of SENP7 and CD20 in tumour tissues from the pCR and non-pCR groups. Scale bars, 50 μm. **(F)** Representative immunohistochemical staining of SENP7 in tumour tissues from the pCR and non-pCR groups. Scale bars for 20× figures are 100 μm, and those for 40× figures are 50 μm. **(G)** Representative multiplex immunofluorescence staining of IL10 and CD20 in tumour tissues from the pCR and non-pCR groups. Scale bars, 50 μm. **(H)** Representative immunohistochemical staining of IL10 in tumour tissues from the pCR and non-pCR groups. Scale bars for 20× figures are 100 μm, and those for 40× figures are 50 μm.

Flow cytometry analysis revealed that knockdown and overexpression of SENP7 significantly affected interleukin-10 (IL-10) expression in primary mouse B cells (Fig. 3A-B and Fig. S3). C₁₂FDG is a fluorogenic substrate commonly used to detect cellular senescence by measuring β-galactosidase activity. Flow cytometric detection of C₁₂FDG revealed that both knockdown and overexpression of SENP7 significantly affected primary mouse B-cell senescence (Fig. 3C-D). Immunohistochemistry and multiplex immunofluorescence confirmed the downregulation of SENP7 and IL10 expression in CD20^+^ cells in the pCR group (Fig. 3E-H).

### High coexpression of SENP7 and SIRT1, with SENP7 activating SIRT1 through deSUMOylation

**Figure 4:**
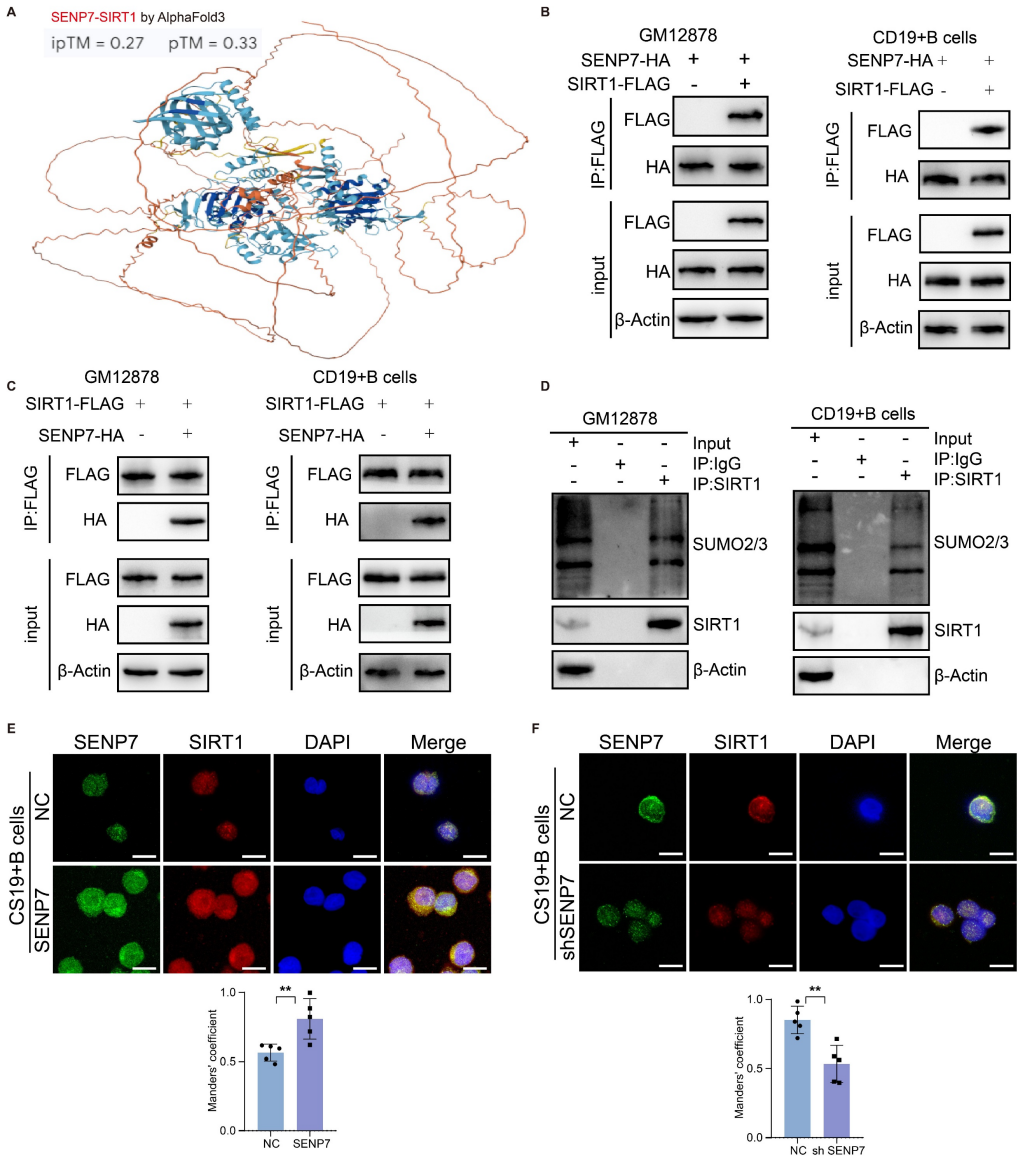
** High coexpression of SENP7 and SIRT1, with SENP7 activating SIRT1 through deSUMOylation. (A)** Molecular docking analysis showing a potential protein‒protein interaction between SENP7 and SIRT1, with an ip^TM^ score of 0.27 and a p^TM^ score of 0.33. **(B-C)** Coimmunoprecipitation (coIP) analysis of the interaction between SENP7 and SIRT1 in GM12878 and primary mouse B cells. **(D)** Endogenous SIRT1 from GM12878 cells and primary mouse B cells was immunoprecipitated using an anti-SIRT1 antibody and subsequently analysed by immunoblotting with the specified antibodies. **(E-F)** Cellular immunofluorescence analysis showing the colocalization of SENP7 and SIRT1 in GM12878 cells. SENP7 and SIRT1 were stained with specific antibodies, and the nuclei were counterstained with DAPI. Representative images are shown along with quantification of fluorescence intensity.

To explore the mechanism through which SENP7 inhibits cellular senescence and induces Breg differentiation, pancancer proteomic sequencing was performed to identify downstream proteins that are positively correlated with SENP7. Among the downstream candidates (Fig. S4A-F), POLR2A (Fig. S4B), CDKN2AIP (Fig. S4C), sirtuin-1 (SIRT1) (Fig. S4D), GABPB1 (Fig. S4D), and TBX21 (Fig. S4F) were markedly coexpressed with Bregs (B-cell cluster 4). Molecular docking revealed SIRT1 and SENP7 as the highest-affinity protein pair (Fig. 4A & Fig. S5A-E), indicating a pivotal mechanistic link in Bregs. Coimmunoprecipitation (coIP) verified this interaction (Fig. 4B-C).

To determine whether SIRT1 undergoes SUMOylation, we evaluated the interaction between SUMO2/3 and endogenous SIRT1 in GM12878 and primary mouse B cells (Fig. 4D). Next, we performed cellular SUMOylation assays by coexpressing tagged SUMO, SIRT1, and SENP7 and compared the SIRT1 SUMOylation levels under NC and shSENP7 conditions, which revealed an increase in SIRT1 SUMOylation upon SENP7 knockdown (Fig. S6). To further validate the association between SENP7 and SIRT1, cellular immunofluorescence analysis was performed, revealing their colocalization and positive correlation with respect to their expression levels (Fig. 4E-F).

### SENP7 promotes IL-10 expression and activates anti-senescence pathways through deSUMOylation-mediated activation of SIRT1

**Figure 5:**
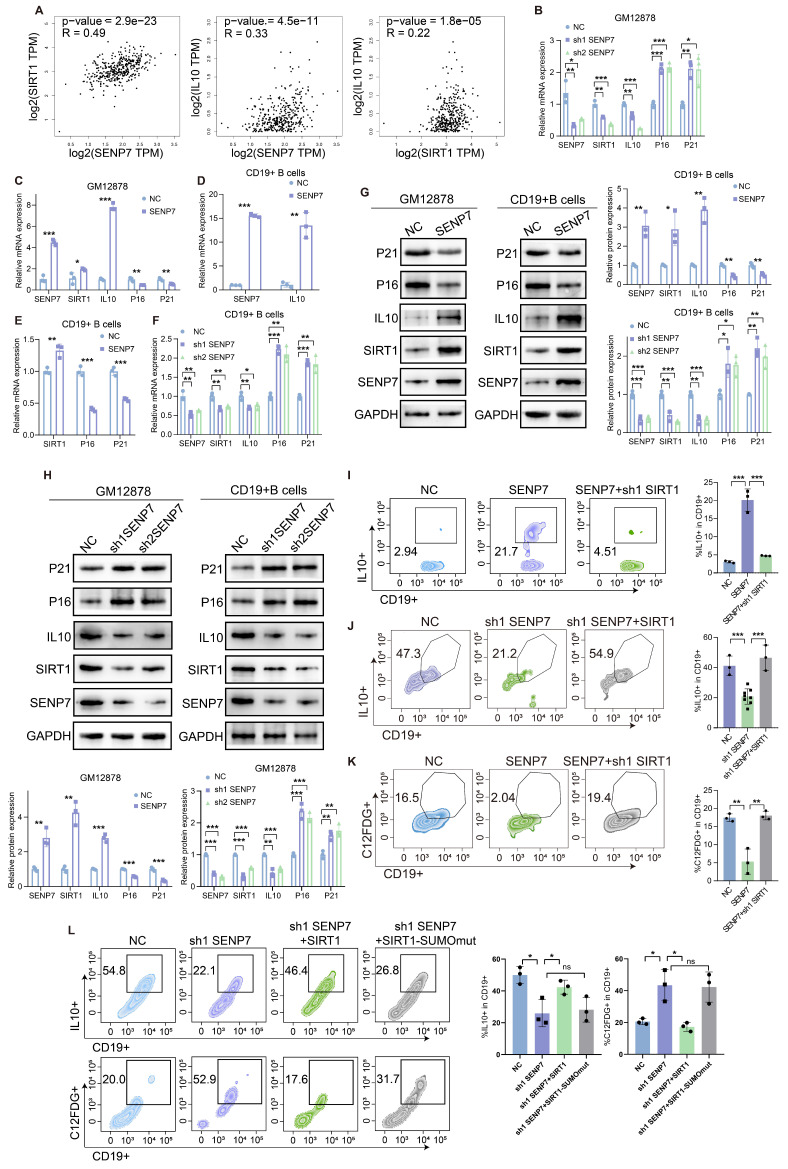
** SENP7 promotes IL-10 expression and activates anti-senescence pathways through deSUMOylation-mediated activation of SIRT1. (A)** Scatter plots showing the pairwise correlations among SENP7, SIRT1, and IL-10 expression levels. Correlation coefficients and statistical significance were determined using Pearson's correlation analysis. **(B-F)** Relative mRNA expression levels of SIRT1, IL-10, P16 and P21 following SENP7 knockdown or overexpression. Data are shown for GM12878 and primary mouse B cells. **(G)** Protein expression levels of SIRT1, IL-10, p16, and p21 following SENP7 overexpression. **(H)** Protein expression levels of SIRT1, IL-10, p16, and p21 following SENP7 knockdown. **(I)** IL-10 expression in primary mouse B cells following SENP7 overexpression, with or without SIRT1 knockdown. **(J)** IL-10 expression in primary mouse B cells following SENP7 knockdown, with or without SIRT1 overexpression. Representative flow cytometry plots and quantification are shown. **(K)** C₁₂FDG fluorescence was measured in primary mouse B cells following SENP7 overexpression, with or without subsequent SIRT1 knockdown. Representative flow cytometry plots and quantification are shown. **(L)** IL-10 expression and C₁₂FDG fluorescence in primary mouse B cells following SENP7 knockdown and rescue with either wild-type (WT) SIRT1 or a deacetylase-deficient (H363Y) SIRT1 mutant. Representative flow cytometry plots and quantification are shown.

Correlation analysis revealed an association between SENP7, SIRT1 and IL10 (Fig. 5A). QPCR and western blot analyses revealed that overexpression of SENP7 upregulated the expression of SIRT1 and IL-10 in GM12878 and primary mouse B cells but downregulated the expression of the senescence markers p16 and p21. Conversely, SENP7 knockdown resulted in the opposite pattern (Fig. 5B-H). Flow cytometry analysis revealed that although overexpression or knockdown of SENP7 modulated IL-10 expression in primary mouse B cells, subsequent knockdown or overexpression of SIRT1 reversed this effect (Fig. 5I-J). Similarly, flow cytometry analysis revealed that although overexpression of SENP7 reduced C₁₂FDG fluorescence in primary mouse B cells, subsequent knockdown or overexpression of SIRT1 reversed this effect (Fig. 5K and Fig. S7). To further determine whether the enzymatic activity of SIRT1 is required for Breg differentiation and senescence inhibition, we performed rescue experiments using a deacetylase-deficient SIRT1 mutant. Notably, unlike wild-type SIRT1, the deacetylase-deficient mutant failed to restore the effects of SENP7 knockdown on IL-10 production and the expression of senescence-associated markers in B cells (Fig. 5L).

### Overexpression of SENP7 promotes Breg differentiation and contributes to CD8⁺ T-cell exhaustion in the tumour immune microenvironment, which can be reversed by SIRT1 knockdown

**Figure 6:**
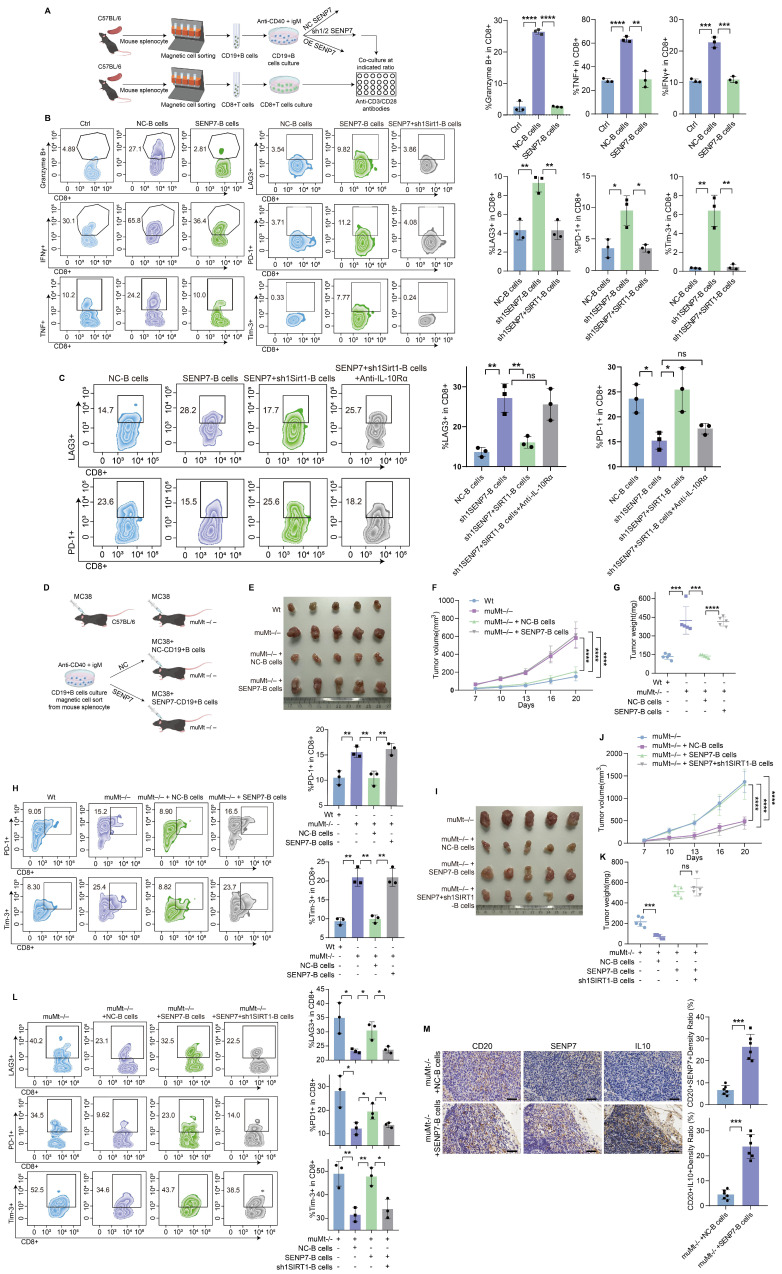
** Overexpression of SENP7 promotes Breg differentiation and contributes to CD8⁺ T-cell exhaustion in the tumour immune microenvironment, which can be reversed by SIRT1 knockdown. (A)** Schematic overview of the experimental design for the B-cell and CD8⁺ T-cell coculture system. **(B)** Flow cytometry analysis of effector and exhaustion markers in CD8⁺ T cells under the indicated coculture conditions: Ctrl, cocultured with control B cells, SENP7-overexpressing B cells, or SENP7-overexpressing B cells with SIRT1 knockdown. Left: representative flow cytometry plots; right: corresponding quantification. **(C)** Flow cytometry analysis of effector function and exhaustion markers in CD8⁺ T cells cocultured with B cells from the indicated groups in the presence or absence of IL-10R blockers. Left: representative flow cytometry plots; right: corresponding quantification. **(D)** Schematic illustration of the adoptive transfer of B cells into muMt^-/-^ mice in a subcutaneous tumour model. **(E)** Representative images of subcutaneous tumours from the following treatment groups: Wt, muMt^-/-^ without B cells, muMt^-/-^ plus NC-B cells, and muMt^-/-^ plus SENP7-overexpressing B cells. **(F)** Tumour volume growth curves over time. **(G)** Tumour weights measured on the day of sacrifice. **(H)** Flow cytometry analysis of tumour-infiltrating lymphocytes showing the expression levels of exhaustion markers (PD-1 and TIM-3) on CD8^+^ T cells. **(I)** Representative images of subcutaneous tumours from the following treatment groups: muMt^-/-^ without B cells, muMt^-/-^ plus NC-B cells, muMt^-/-^ plus SENP7-overexpressing B cells, and muMt^-/-^ plus SENP7-overexpressing B cells with SIRT1 knockdown. **(J)** Tumour volume growth curves over time. **(K)** Tumour weights measured on the day of sacrifice. **(L)** Flow cytometry analysis of tumour-infiltrating lymphocytes showing the expression levels of exhaustion markers (LAG3, PD-1, TIM-3) on CD8^+^ T cells. **(M)** Immunohistochemical analysis of IL-10 expression in CD20⁺ B cells from tumours of mice receiving adoptive transfer of NC-B or SENP7-overexpressing B cells. The tumour sections were stained for CD20 and IL-10, and representative images are shown. Scale bars for 40× figures are 50 μm.

Flow cytometry analysis revealed that coculture with B cells enhanced CD8⁺ T-cell effector function, whereas SENP7 overexpression in B cells suppressed this effect and increased exhaustion marker expression. Notably, SIRT1 knockdown reversed these SENP7-mediated effects (Fig. 6A-B and Fig. S3). Knockdown of SENP7, either alone or in combination with SIRT1 overexpression, yielded consistent results (Fig. S8). To determine whether the immunosuppressive effect is solely mediated by the SENP7-SIRT1 axis, B cells from different groups were cocultured with CD8⁺ T cells in the presence or absence of IL-10R blockers. Treatment with IL-10R blockers further restored effector function and reduced exhaustion marker expression, indicating that the SENP7-SIRT1 axis mediates immunosuppression in part through B-cell-derived IL-10 (Fig. 6C). The subcutaneous tumour model further demonstrated that adoptive transfer of B cells led to a marked reduction in tumour growth, whereas overexpression of SENP7 in B cells abrogated this effect. Moreover, additional knockdown of SIRT1 in SENP7-overexpressing B cells suppressed tumour growth (Fig. 6D-G & 6I-K). Consistently, flow cytometry analysis of tumour-infiltrating lymphocytes from subcutaneous tumours revealed a corresponding expression pattern of exhaustion markers on CD8⁺ T cells (Fig. 6H & 6L). Immunohistochemistry results revealed that compared with CD20⁺ B cells in mice receiving NC-B cells, CD20⁺ B cells in the caecal orthotopic tumours of mice receiving adoptive transfer of SENP7-overexpressing B cells exhibited higher levels of IL-10 expression (Fig. 6M).

### Targeting SENP7 in B cells enhances the efficacy of anti-PD-1 immunotherapy

**Figure 7:**
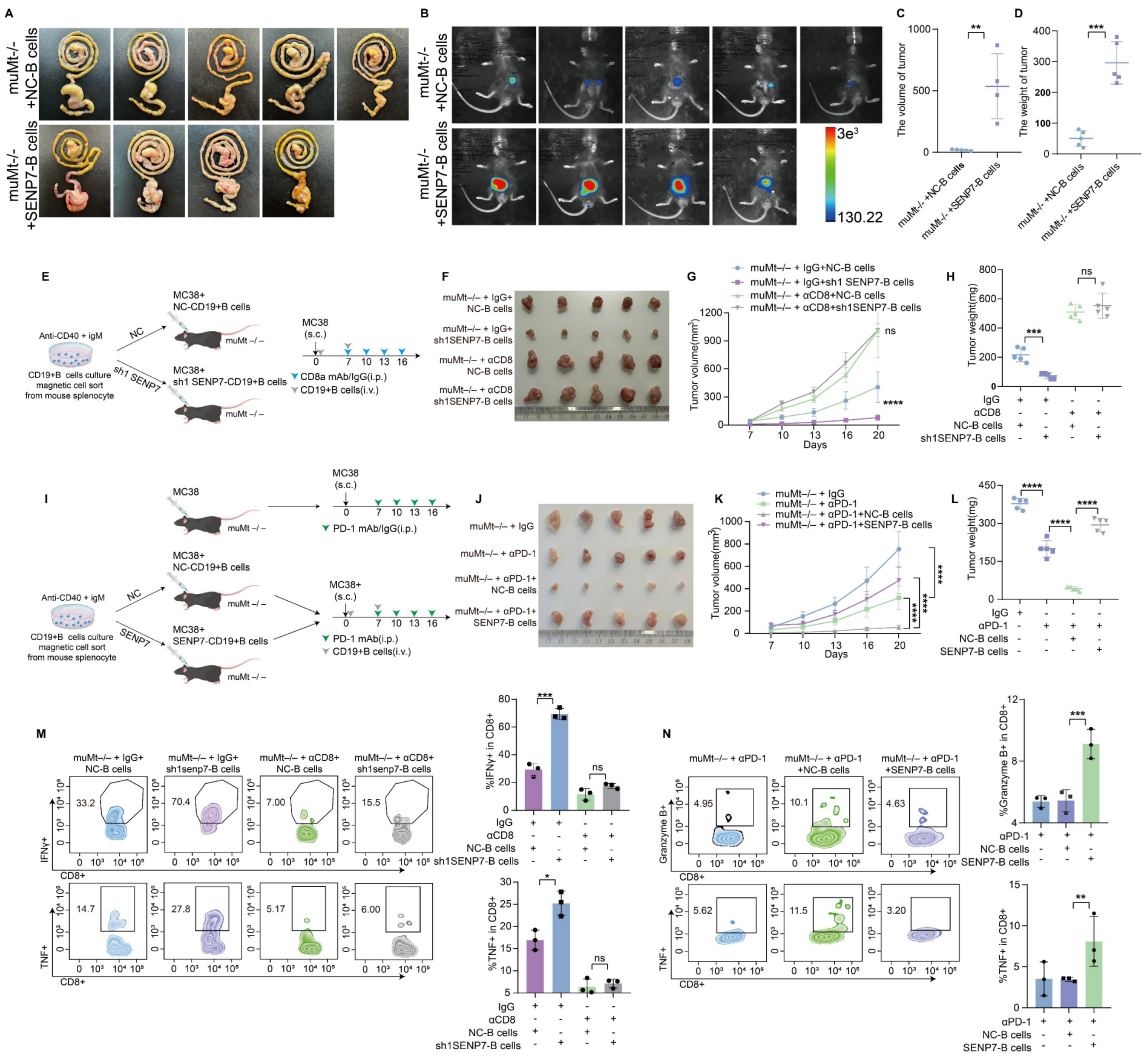
** Targeting SENP7 in B cells enhances the efficacy of anti-PD-1 immunotherapy. (A)** Representative macroscopic images of caecal tumours from muMt^-/-^ mice following adoptive transfer of either control or SENP7-overexpressing B cells. **(B)** Representative bioluminescence images depicting the tumour burden in each group at the time of sacrifice. **(C)** Tumour volumes measured on the day of sacrifice. **(D)** Tumour weights measured on the day of sacrifice. **(E)** Schematic diagram of the experimental design. **(F)** Representative macroscopic images of tumours from the following groups: muMt^-/-^ + IgG + NC-B cells, muMt^-/-^ + IgG + sh1 SENP7-B cells, muMt^-/-^ + αCD8 + NC-B cells, and muMt^-/-^ + αCD8 + sh1 SENP7-B cells. **(G)** Tumour growth curves measured over time for each group. **(H)** Tumour weights measured on the day of sacrifice for the indicated groups. **(I)** Schematic diagram of the experimental design. **(J)** Representative macroscopic images of tumours from the following groups: muMt^-/-^ + IgG, muMt^-/-^ + αPD1, muMt^-/-^ + αPD1 + NC-B cells, and muMt^-/-^ + αPD1 + SENP7-B cells. **(K)** Tumour growth curves measured over time for each group. **(L)** Tumour weights measured on the day of sacrifice for the indicated groups. **(M-N)** Flow cytometry analysis of effector function expression levels in tumour-infiltrating CD8^+^ T cells from each treatment group.

To elucidate the role of SENP7 in the tumour microenvironment (TME) of colorectal cancer CRC, we further employed an *in situ* tumour model in which adoptive transfer of B cells overexpressing SENP7 significantly accelerated tumour growth (Fig. 7A-D). To determine whether the tumour-suppressive effect of SENP7 knockdown in B cells was mediated by CD8⁺ T cells, we employed a CD8⁺ T-cell depletion strategy involving the use of anti-CD8 antibodies in a murine model. Depletion of CD8⁺ T cells eliminated the inhibitory effect of SENP7-deficient B cells on tumour progression (Fig. 7E-H). We next evaluated the therapeutic effect of combining B-cell adoptive transfer with anti-PD-1 monoclonal antibody treatment in MC38 tumour-bearing mice. Compared with either treatment alone, the combination therapy led to reduced tumour growth. However, this antitumour effect was negated when SENP7 was overexpressed in the transferred B cells (Fig. 7I-L). To assess the long-term efficacy and safety of combining B-cell adoptive transfer with anti-PD-1 monoclonal antibody therapy, we first employed an *in situ* tumour model. Compared with either treatment alone, the combination therapy improved survival, which was further enhanced when SENP7 was knocked down in the transferred B cells (Fig. S9A-D). In a prolonged subcutaneous tumour model (approximately 35 days), the inhibitory effect of anti-PD-1 monotherapy on tumour growth gradually decreased over time, whereas B-cell adoptive transfer restored tumour control, with further enhancement upon SENP7 knockdown in the transferred B cells (Fig. S9E-G). Conversely, SENP7 overexpression in transferred B cells abrogated the therapeutic benefit of the combination treatment (Fig. S9H-J). Consistently, flow cytometry analysis of tumour-infiltrating lymphocytes from subcutaneous tumours revealed a corresponding expression pattern of effector function markers on CD8⁺ T cells (Fig. 7M-N).

## Discussion

Some current studies on tumour immunotherapy mechanisms focus on how tumour cells modulate the immune microenvironment^22^,^23^. Although mechanistic studies in tumour immunotherapy increasingly emphasize immune cells^24^-^26^, the specific mechanisms through which B-cell subsets regulate anti-PD-1 efficacy remain poorly understood. Regulatory B-cell (Breg)-derived IL-10 is a crucial immunosuppressive factor that suppresses CD8⁺ T-cell function in the tumour microenvironment (TME), facilitating immune evasion. We identify sentrin-specific protease 7 (SENP7) as a potential therapeutic target on B cells, where its inhibition restrains Breg differentiation and may represent a novel strategy to counteract tumour immune escape.

Senescent-like cells have been shown to possess enhanced immunostimulatory potential under specific conditions^27^,^28^. However, the link between B-cell senescence and tumour immune activation remains unexplored. Our study demonstrated that targeting SENP7 in B cells induces a senescent phenotype and impairs Breg differentiation, thereby enhancing CD8⁺ T-cell-mediated antitumour immunity.

Despite the growing interest in posttranslational modifications in the TME, studies specifically addressing the role of SUMOylation in regulating immune cell function remain limited. This study reveals a previously unrecognized mechanism by which the SUMO-specific protease SENP7 promotes Breg differentiation. Specifically, we demonstrate that SENP7 activates the NAD-dependent protein deacetylase sirtuin-1 (SIRT1) via deSUMOylation in B cells, thereby enhancing the transcriptional production of the immunosuppressive cytokine IL-10.

Our results indicate that inhibiting SENP7 in B cells could serve as a promising strategy to render immunologically “cold” tumours more responsive to immune checkpoint inhibitors. Overall, this study defines the SENP7-SIRT1-IL-10 signalling axis in B cells as the conceptual centrepiece linking deSUMOylation, senescence, and immune modulation, providing a focused mechanistic framework that unifies these interconnected processes. Additionally, by revealing a previously unrecognized link between SUMOylation, cellular senescence, and immune modulation, this study acknowledges that although SENP7 represents a promising therapeutic node, potential challenges such as enzyme redundancy within the SENP family, substrate selectivity, and possible systemic effects should be carefully evaluated before clinical translation. These considerations highlight the need to develop highly specific modulators and B-cell-targeted delivery strategies to minimize off-target effects. This study offers translational insights that may guide the design of combination therapies to overcome resistance to immunotherapy in clinical settings.

## Supplementary Material

Supplementary methods, figures and tables 1-2.

Supplementary table 3.

Supplementary table 4.

Supplementary table 5.

## Abbreviations

CRC: colorectal cancer
ICB: immune checkpoint blockade
dMMR: mismatch repair-deficient
MSI-H: microsatellite instability-high
SENP7: sentrin-specific protease 7
Breg: regulatory B cell
SIRT1: NAD-dependent protein deacetylase sirtuin-1
TME: tumour microenvironment
Tex: exhausted T cells
IL-10: interleukin-10
TGF-β: transforming growth factor-β
ICI: immune checkpoint inhibitor
CAP-TRG: College of American Pathologists Tumor Regression Grading
coIP: coimmunoprecipitation
