# Supplementary material for: A SENP7-SIRT1-IL-10 Axis Driven by DeSUMOylation Promotes Breg Differentiation and Immune Evasion in Colorectal Cancer
Source: Int J Biol Sci. 2026 Jan 1;22(1):111–25. doi: 10.7150/ijbs.118896 (PMC12681700; doi:10.7150/ijbs.118896)
Supplement: Supplementary file 1 — Supplementary methods, figures and tables 1-2. [file ijbsv22p0111s1.pdf]

Supplementary Materials for

A SENP7–SIRT1–IL-10 Axis Driven by DeSUMOylation Promotes Breg Differentiation and Immune Evasion in Colorectal Cancer

Authors: Yuhan Liao[1,2#], Xinghua Zhuo[3#], Yuan Huang[4#], Huimeng Xu[1,2], Zhe Hao[1,2], Lanhui Huang[1,2], Haoxuan Zheng[3*], Jun Zhou[1,2,5*]

Correspondence to: Junzhou: jhzhou@smu.edu.cn & chuhang127@163.com;

Haoxuan Zheng: ryan801218@163.com

This PDF file includes:

Supplementary Materials and Methods

Figures. S1 to S9

Tables S1 to S2

Cell lines and cell culture

GM12878 were purchased from Meisen CTCC (Hangzhou, China). Engineered cell line 293T was purchased from the Cell Bank of Type Culture Collection of the Chinese Academy of Sciences (Shanghai, China). All cell lines were cultured in RPMI 1640 medium (KeyGEN BioTECH, Jiangsu, China) supplemented with 10% fetal bovine serum (FBS) (Gibco-BRL, Invitrogen, Paisley, UK). Cells were maintained in a humidified atmosphere containing 5% CO2 at 37°C. All cell lines used in this study tested negative for mycoplasma and were authenticated by short tandem repeat (STR) profiling within four years. All cell line experiments were performed within six months of thawing or cell collection. Cells were transfected with plasmid vectors using Lipofectamine ® 3000 reagent (Thermo Fisher Scientific, USA) according to the manufacturer's protocol.

Primers, shRNAs

qPCR primers and shRNAs are shown in supplemental table S1.

Plasmid construction and lenti-virus transduction

All lentiviral vectors and control vectors were purchased from Tsingke (Guangzhou, China). To generate lentiviral supernatant, the plasmids containing the genetic information of the lentivirus, as well as the packaging vector psPAX2 (Addgene, 12260) and envelope vector PMD2.G (Addgene, 12259), are co-transfected into 293T cells. After 12 h of transfection, the medium was replaced, and the virus-containing supernatant was collected 48 h later. The collected supernatant was filtered through a 0.45 μm PVDF filter with small pores (Millipore, Darmstadt, Germany). And then, 8

µg/mL Polybrene (Sigma-Aldrich, 107689), a transduction enhancer, was added to the filtered supernatant prior to infection of the target cells.

Immunohistochemistry (IHC)

Immunohistochemistry was performed as previously described[1], to investigate the expression of proteins in clinical specimens and mouse subcutaneous tumor tissue. The tissue sections were incubated overnight using primary antibodies (Primary antibodies used are listed in supplemental table S2). Mayer's haematoxylin was used for nuclear counterstaining. For IHC staining, we used a semi-quantitative method, which was calculated by an established semi-quantitative assessment of both the intensity of staining and the percentage of positive cells following an established procedure. The staining intensity in the malignant cell was scored as 0, 1, 2, or 3 for the presence of negative, weak, intermediate, or strong staining, respectively. As well as, the expression ratio was scored as 1 (0-25%), 2 (26-50%), 3 (51-75%), and 4 (76-100%). The final score for immunohistochemistry is staining intensity multiplied by area, for a total of 12 points. In this study, we define a score greater than or equal to 6 as a "high expression", and a score less than 6 as a "low expression". The slides were reviewed and scored by at least two, and usually three, certified anatomic pathologists. The 5% of discrepancies were resolved through simultaneous re-evaluation. The Pearson's chi-squared test (χ^2) was used to determine the significance of the correlation.

Western Blot

Total protein extraction was performed with RIPA lysis buffer supplemented with a

protease inhibitor cocktail. And protein was quantified by the BCA Protein Assay Kit (Pierce, KeyGEN BioTECH, Jiangsu, China). After denaturation, proteins were separated by SDS-PAGE gel and transferred to the PVDF membrane (Millipore, Darmstadt, Germany). A Tris buffer containing 0.1% Tween-20 and 5% non-fat dry milk was used to block the membrane at RT for 1 h. The membrane was incubated overnight with antibodies (Primary antibodies used are listed in supplemental table S2.).Blots were then washed and incubated for 1 h at RT with horseradish peroxidase (HRP)-labeled secondary antibodies (anti-rabbit IgG or anti-mouse IgG, Cell Signaling Technology, 1: 10,000). The protein was detected by an ECL chemiluminescence solution (Epizyme Biomedical Technology Co., Ltd, Shanghai, China) and finally visualized using an enhanced chemiluminescence detection system (Tennon5200, Shanghai, China).

Co-immunoprecipitation (Co-IP) Assay

Cells were lysed in a non-denaturing lysis buffer containing protease and phosphatase inhibitors. The lysates were pre-cleared with control IgG and protein A/G agarose beads, then incubated with specific primary antibodies overnight at 4° C with gentle rotation. Protein complexes were captured using protein A/G beads, followed by extensive washing with lysis buffer to remove non-specific interactions. Bound proteins were eluted by boiling in SDS-PAGE loading buffer and analyzed by Western blotting.

RNA isolation, reverse transcription, and quantitative PCR (qPCR)

Total RNA was extracted from cells and tissue using the Trizol reagent (Invitrogen,

Carlsbad, California). And then 1 μg RNA was used to reverse transcribe cDNA using the Evo M-MLV Reverse Transcription Kit (Accurate Biotechnology Co., Ltd, Hunan, China). QPCR analyses were carried out on retrotranscribed cDNAs with the SYBR® Geen Pro Taq HS qPCR kit (Accurate Biotechnology Co., Ltd, Hunan, China). Expression was measured on an Applied Biosystems 7500 Fast Real-Time PCR system. Experiments were performed at least three times, with duplicate replicates. The quantification is based on the $2^{-\Delta\Delta Ct}$ method using the housekeeping gene GAPDH as a normalizer.

Statistical analyses

The figures and graphical elements in this manuscript were created and compiled using Figdraw (https://www.figdraw.com/) , BioGD (Phttps://biogdp.com/) and Adobe Illustrator 2023 (Adobe, San Jose, CA, USA). The results were analyzed using SPSS statistical software (version 24.0; SPSS, Inc., Chicago, IL, USA) and GraphPad Prism 8 software (GraphPad Software, Inc., CA, USA). Statistical tests included Student's t-tests (paired or unpaired), one-way and two-way analysis of variance (ANOVA). Statistical significance was set at $P < 0.05$. * $P < 0.05$, ** $P < 0.01$, *** $P < 0.001$, ns not significant.

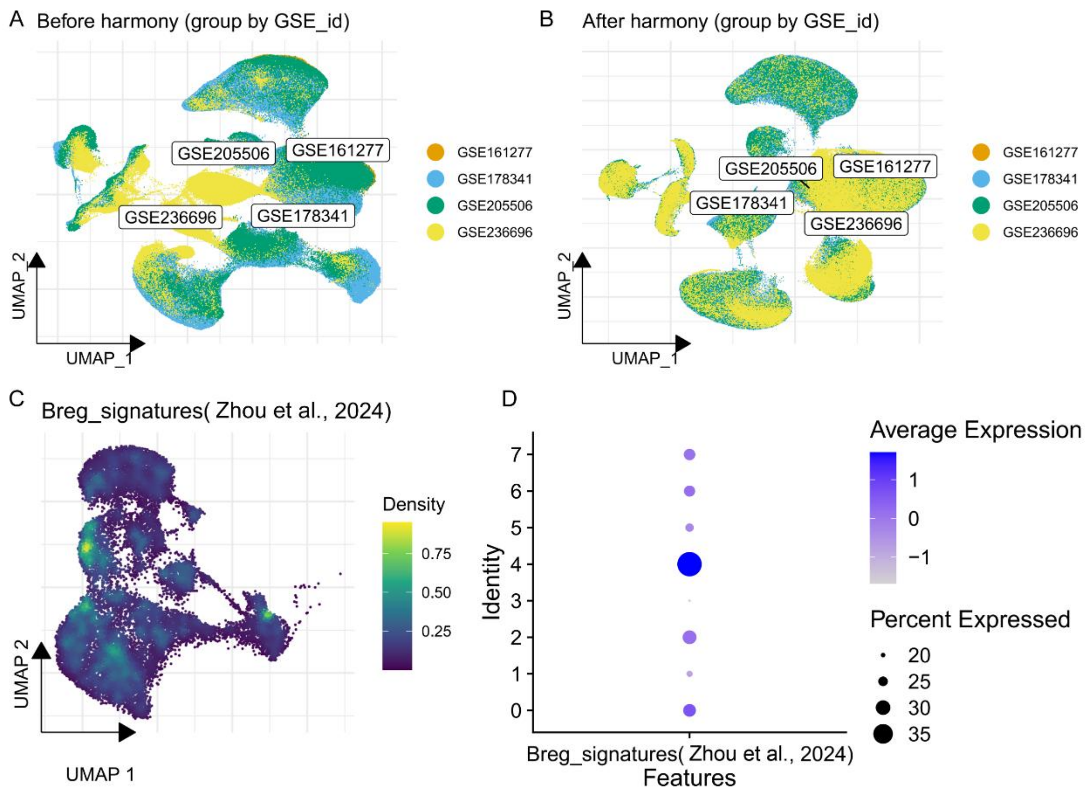

Figure S1. Integration of multi-dataset single-cell transcriptomes and identification of Breg signatures. (A) UMAP visualization of cells from 4 GEO datasets before harmony integration, grouped by dataset ID. **(B)** UMAP visualization after harmony correction showing effective removal of batch effects and improved dataset mixing. **(C)** Density distribution of Breg signatures (Zhou et al., 2024) projected on the integrated UMAP plot. **(D)** Dot plot showing expression pattern of Breg signatures (Zhou et al., 2024) across different B-cell clusters.

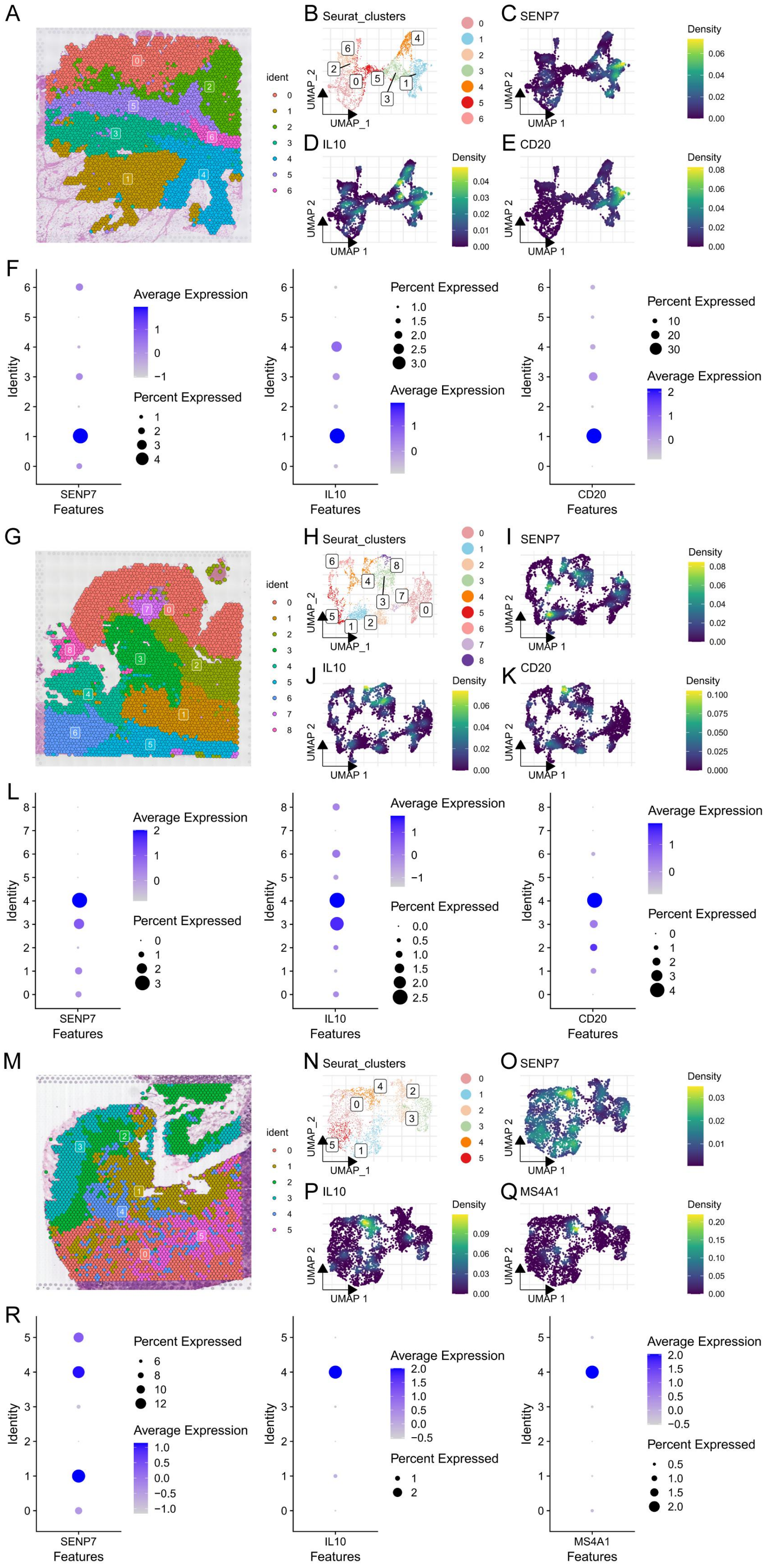
A
ident
B Seurat_clusters
UMAP_1
UMAP_2
C SENP7
Density
D IL10
E CD20
UMAP 1
UMAP 2
F
Identity
Average Expression
Percent Expressed
SENP7
IL10
CD20
Features
G
H Seurat_clusters
I SENP7
J IL10
K CD20
L
M
N Seurat_clusters
O SENP7
P IL10
Q MS4A1
R
MS4A1

Figure S2. Spatial co-expression of SENP7, IL10, and CD20. (A–F) Spatial mapping and gene-expression patterns in section GSM7089856. **(A)** Spatial distribution of Seurat-defined clusters overlaid on tissue morphology. **(B)** UMAP visualization showing 7 transcriptional clusters. **(C–E)** Spatial density plots showing SENP7, IL10, and CD20 expression, respectively. **(F)** Dot plots showing expression levels and proportions across clusters. **(G–L)** Spatial mapping and gene-expression patterns in section GSM7089858. **(G)** Spatial distribution of Seurat-defined clusters overlaid on tissue morphology. **(H)** UMAP visualization showing 9 transcriptional clusters. **(I–K)** Spatial density plots of SENP7, IL10, and CD20 expression. **(L)** Dot plots showing expression levels and proportions across clusters. **(M–R)** Spatial mapping and gene-expression patterns in the colorectal cancer section ST-colon1 derived from *Wu et al.*, *Cancer Discovery* (2022), 12(1): 134–153 (DOI 10.1158/2159-8290.CD-21-0316). **(M)** Spatial distribution of Seurat-defined clusters overlaid on tissue morphology. **(N)** UMAP visualization showing 6 transcriptional clusters. **(O–Q)** Spatial density plots of SENP7, IL10, and MS4A1 expression. **(R)** Dot plots showing expression levels and proportions across clusters.

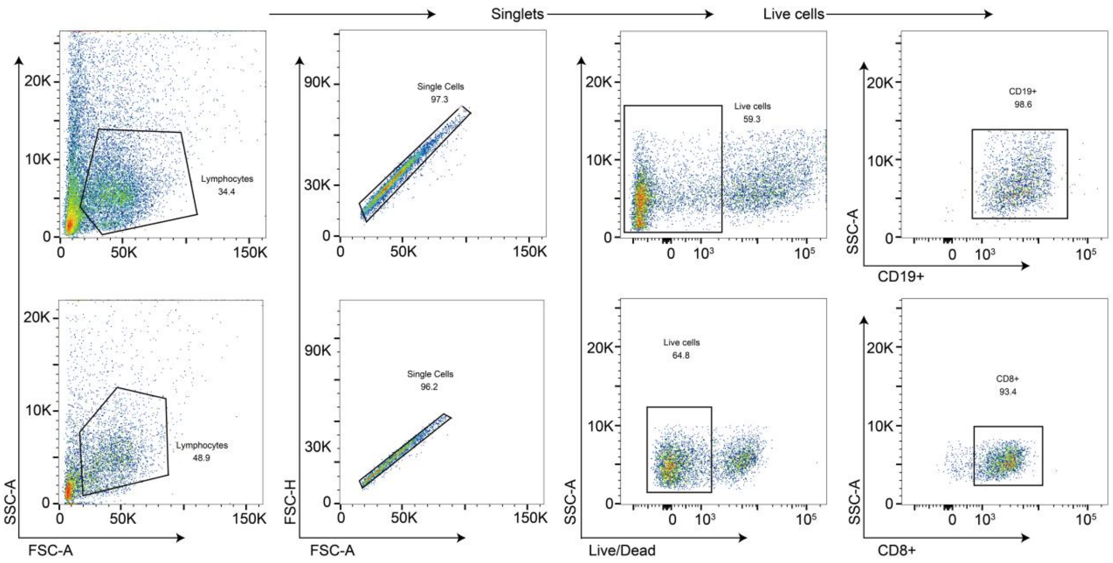

Figure S3. Gating strategies for flow cytometric analysis of primary B cells and primary $CD8^+$ T cells.

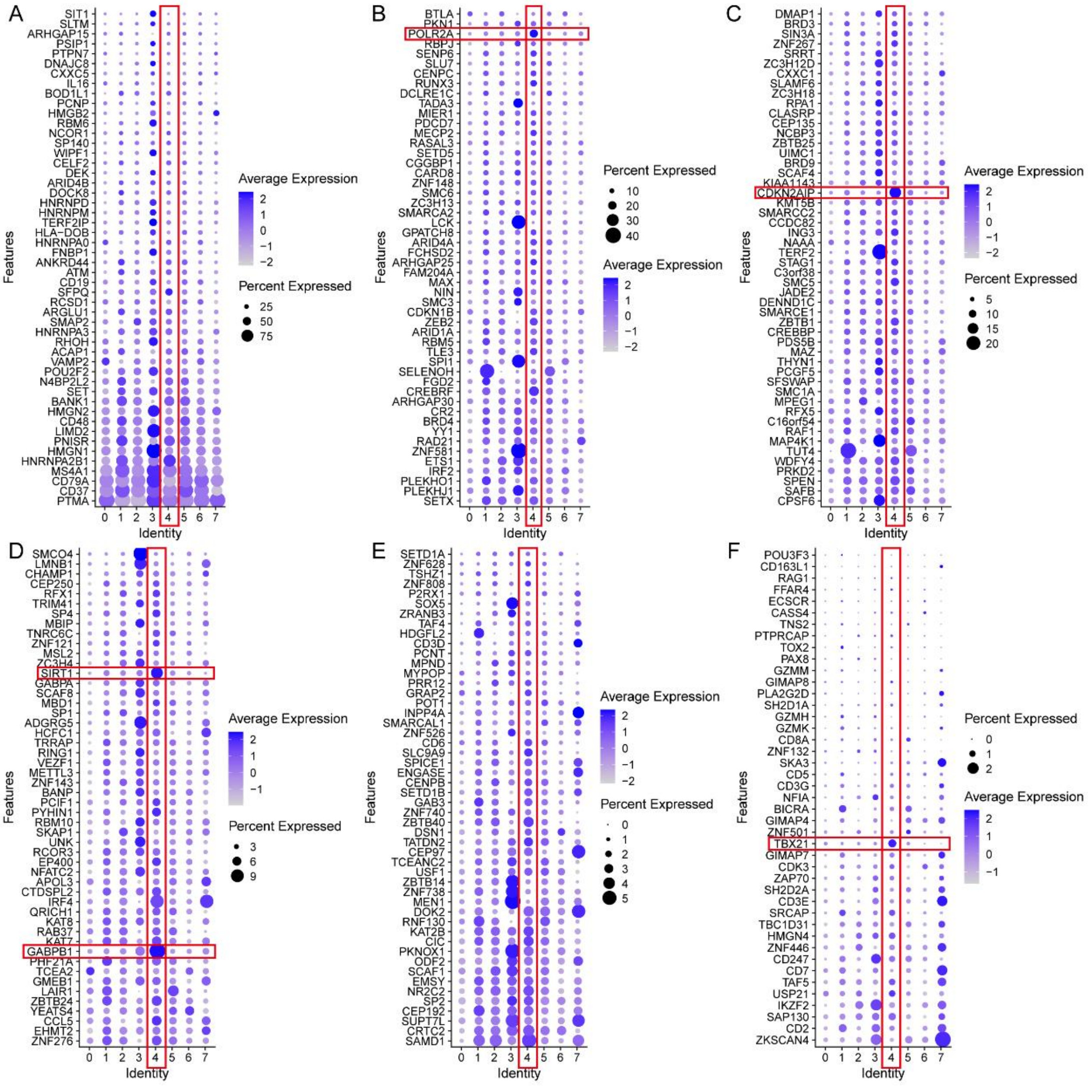

Figure S4. Projection of SENP7 positively correlated proteins onto B-cell clusters. **(A-F)** Dot plots showing the expression of SENP7-associated proteins across different B-cell clusters. The B-cell cluster 4 was annotated as Breg. Proteins positively correlated with SENP7 (correlation coefficient >0.2 and p <0.001) were projected onto B-cell clusters. Red boxes highlighted proteins (POLR2A, CDKN2AIP, SIRT1, GABPB1, and TBX21) that, similar to SENP7, exhibit co-expression patterns within Bregs (B-cell cluster 4).

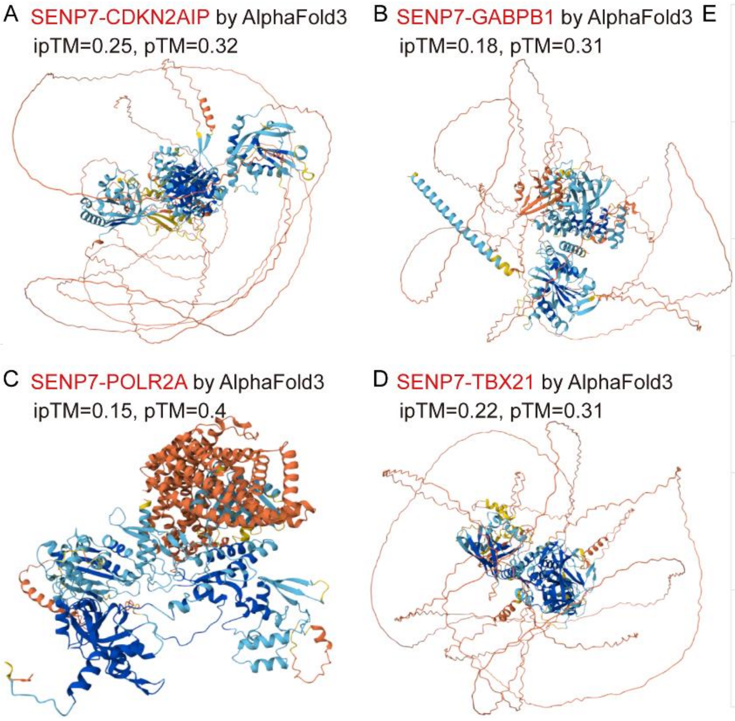

E

Protein Pair	iPTM	pTM	iPTM + pTM
SENP7-SIRT1	0.27	0.33	0.6
SENP7-CDKN2AIP	0.25	0.32	0.57
SENP7-POLR2A	0.15	0.4	0.55
SENP7-TBX21	0.22	0.31	0.53
SENP7-GABPB1	0.18	0.31	0.49

Figure S5. Molecular docking prediction of SENP7-protein interactions by AlphaFold3. (A-D) Predicted complex structures of SENP7 with CDKN2AIP, GABPB1, POLR2A, and TBX21, respectively. **(E)** Summary table showing interface-predicted TM-score (ipTM), predicted TM-score (pTM), and their combined values (ipTM + pTM) for SENP7 with 5 candidate proteins, highlighting SIRT1 as the strongest predicted interactor.

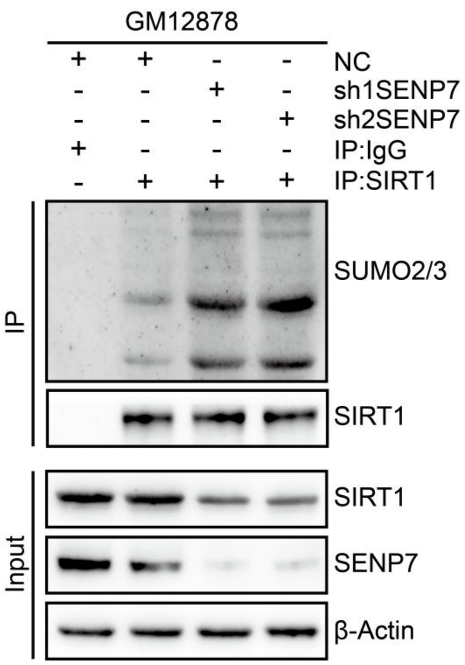

Figure S6. Endogenous SIRT1 from GM12878 cells and primary mouse B cells, with or without SENP7 knockdown, was immunoprecipitated using an anti-SIRT1 antibody, followed by immunoblotting with the indicated antibodies.

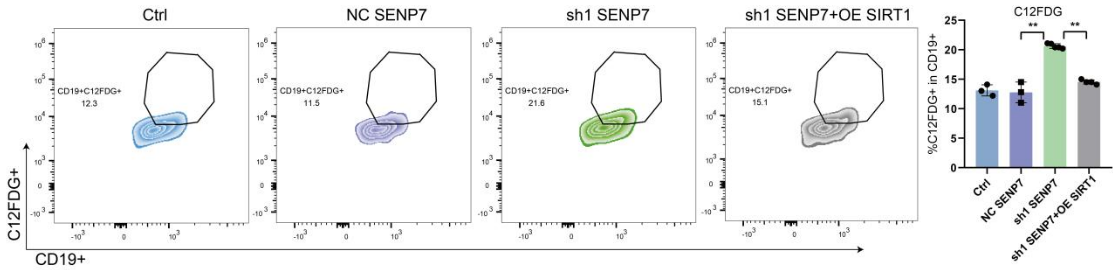

Figure S7. C_{12}FDG fluorescence was measured in primary mouse B cells following SENP7 knockdown, with or without subsequent SIRT1 overexpression. Representative flow cytometry plots and quantification are shown.

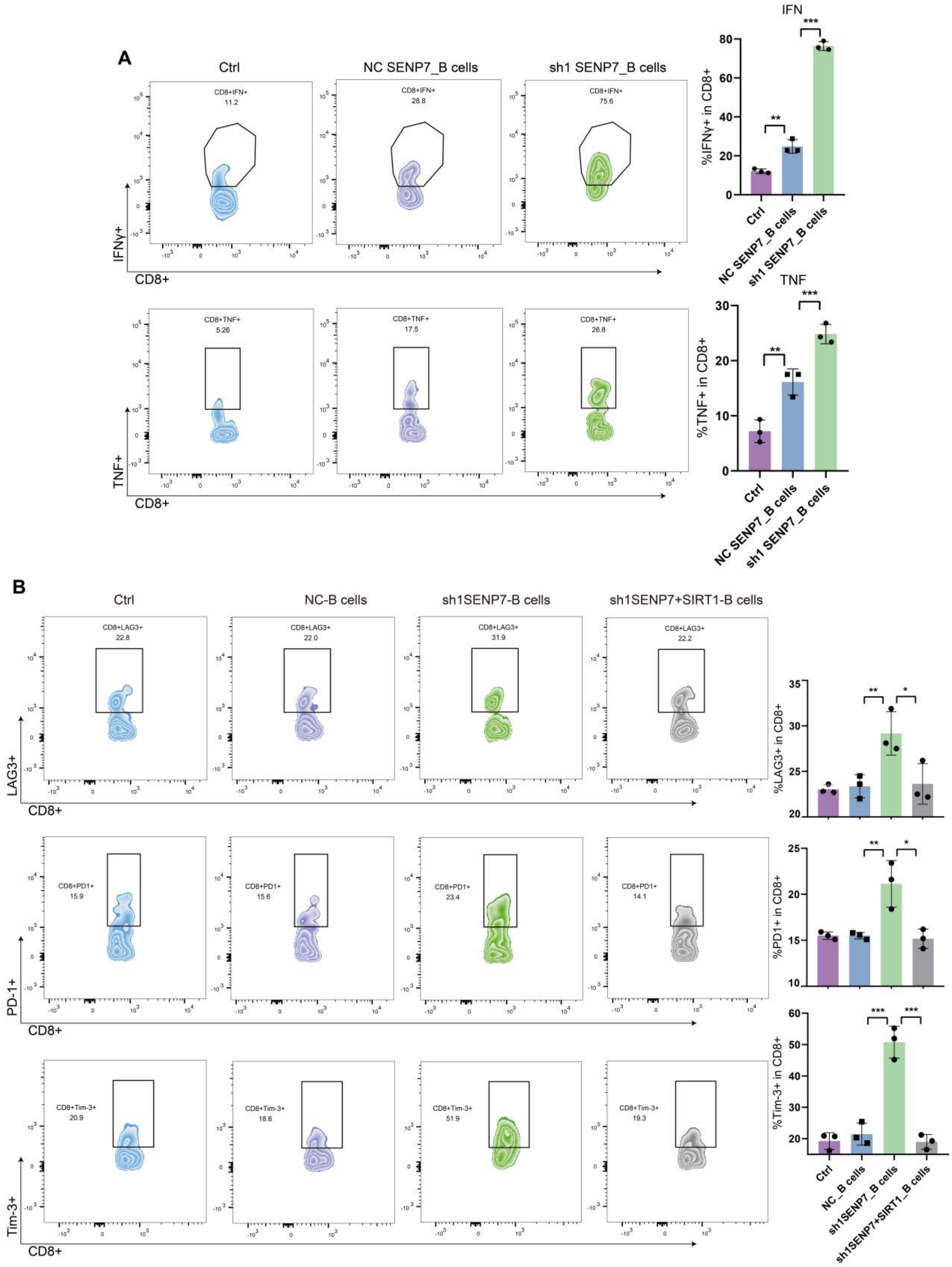

Figure S8. **(A)** Flow cytometry analysis of function markers (IFNγ and TNF) on CD8^{+} T cells. **(B)** Flow cytometry analysis of exhaustion markers (PD-1, LAG3, and Tim-3) on CD8^{+} T cells.

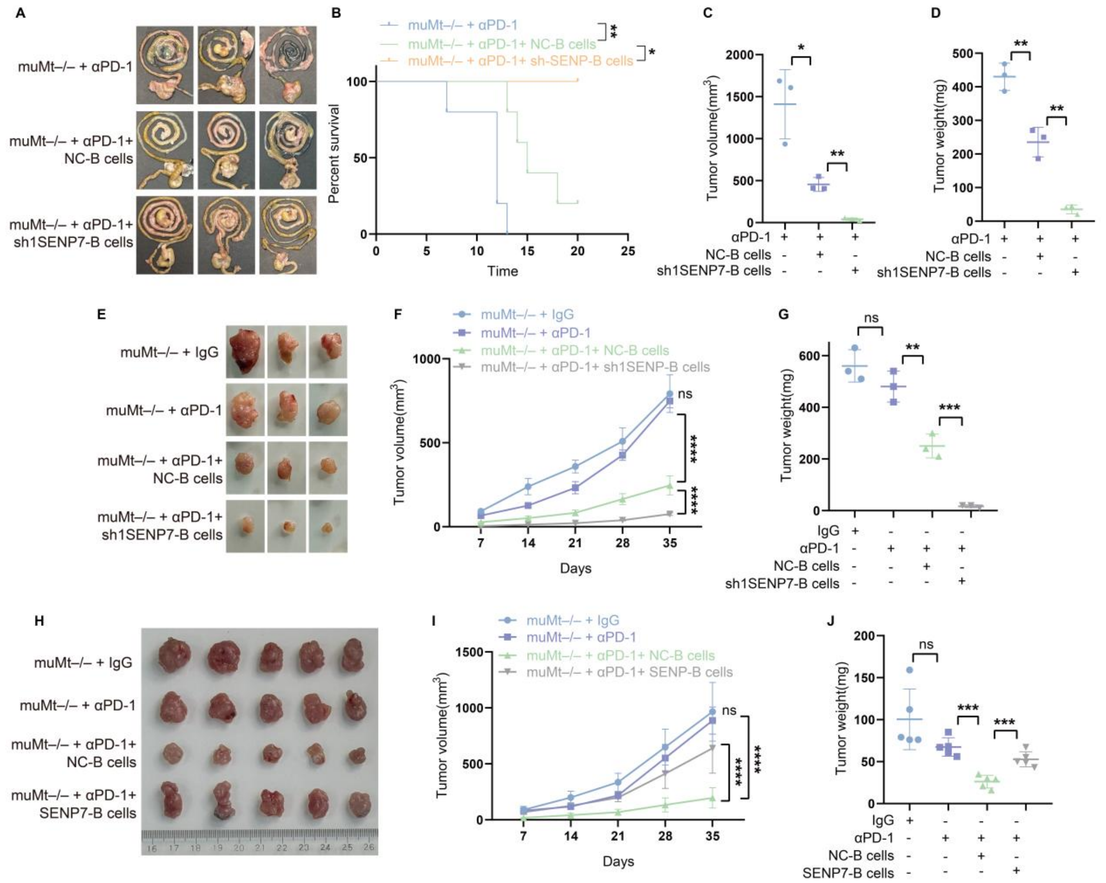

Figure S9. **(A)** Representative macroscopic images of tumors from the following groups: muMt$^{-/-}$ + αPD1, muMt$^{-/-}$ + αPD1 + NC-B cells, muMt$^{-/-}$ + αPD1 + sh1SENP7-B cells. **(B)** Kaplan – Meier survival curves of tumor-bearing mice receiving adoptive transfer of primary mouse B cells with or without SENP7 knockdown. Survival differences were analyzed using the log-rank (Mantel – Cox) test. **(C)** Tumor volumes measured on the day of sacrifice for the indicated groups. **(D)** Tumor weights measured on the day of sacrifice for the indicated groups. **(E)** Representative macroscopic images of tumors from the following groups: muMt$^{-/-}$ + IgG, muMt$^{-/-}$ + αPD1, muMt$^{-/-}$ + αPD1 + NC-B cells, muMt$^{-/-}$ + αPD1 + sh1SENP7-B cells. **(F)** Tumor growth curves measured over time for each group. **(G)** Tumor weights measured on the day of sacrifice for the indicated groups. **(H)** Representative

macroscopic images of tumors from the following groups: muMt$^{-/-}$ + IgG, muMt$^{-/-}$ + αPD1, muMt$^{-/-}$ + αPD1 + NC-B cells, muMt$^{-/-}$ + αPD1 + SENP7-B cells. **(I)** Tumor growth curves measured over time for each group. **(J)** Tumor weights measured on the day of sacrifice for the indicated groups.

Supplementary Table 1. Primers and shRNAs used in this study

Name		Sequence
GAPDH	Forward	5'-AGAAGGCTGGGGCTCATTTG-3'
	Reverse	5'-GAGGGGCCATCCACAGTCTTC-3'
SENP7	Forward	5'-TTGTTTATCCTCCACCACCTAC-3'
	Reverse	5'-CTCTGAGCCACTGATAGATCTG-3'
SIRT1	Forward	5'-ACAAAGTTGACTGTGAAGCTGTAC-3'
	Reverse	5'-GTTCATCAGCTGGGCACCTA-3'
P16	Forward	5'-GCTGCCCAACGCACCGAATA-3'
	Reverse	5'-ACCACCAGCGTGTCCAGGAA-3'
P21	Forward	5'-AGGTGGACCTGGAGACTCTCAG-3'
	Reverse	5'-TCCTCTTGGAGAAGATCAGCCG-3'
IL10	Forward	5'-CGCAGTGCAGAAGAGTCGAC-3'
	Reverse	5'-CCCGCTTGAGATCCTGAAATAA-3'
shSENP7-1 target sequence		5'-CAAAGUACCGAGUCGAAUAUU-3'
shSENP7-2 target sequence		5'-GAUAAUGAUCUACGUACUAUU-3'
ShSIRT1-1 target sequence		5'-GTACCGGCATGAAGTGCCTCAGATATTACTCGAGTAATATCTGAGGCACTTCATGTTTTTTG-3'
shSIRT1-2 target sequence		5'-CCGGGCAAAGCCTTTCTGAATCTATCTCGAGATAGATTCAGAAAGGCTTTGCTTTTT-3'
lghm-KO-IF		5'-TGTGCCCATTCCAGGTAAG-3'
lghm-KO-IR		5'-CCAAAGTTCAAGGAGCAAATG-3'

Supplementary Table 2. Antibodies used in this study

Antibodies	SOURCE	CATALOG
Brilliant Violet 605™ anti-mouse CD8a	Biolegend	100743
FITC anti-mouse CD8a	Biolegend	100705
APC anti-mouse IFN-γ	Biolegend	505809
Brilliant Violet 421™ anti-mouse TNF-α	Biolegend	506327
Alexa Fluor 700 anti-mouse Granzyme	Biolegend	372221
Brilliant Violet 605™ anti-mouse CD279 (PD-1)	Biolegend	135219
Brilliant Violet 510™ anti-human CD223 (LAG-3)	Biolegend	369317
PerCP/Cyanine5.5 anti-mouse CD366 (Tim-3)	Biolegend	134011
PerCP anti-mouse CD19	Biolegend	115531
anti-SENP7	immunoway	YT4241
anti-SIRT1	Abcam	ab32441
anti-IL10	Santa Cruz Biotechnology	sc-8438
Anti-CD8 alpha antibody	Abcam	ab4055
Anti-p21 antibody [EPR362]	Abcam	ab109520
Anti-CDKN2A/p16INK4a antibody [EPR24167-43]	Abcam	ab270058
Anti-CD20	Abcam	ab64088
Anti-PRELID1	Invitrogen	PA5-31087

References

1. Liao, Y. et al. Artificial Intelligence for Predicting HER2 Status of Gastric Cancer Based on Whole-Slide Histopathology Images: A Retrospective Multicenter Study. *Adv. Sci.* **12**, 2408451

(2025).
